# Clinical Uses of Botulinum Neurotoxins: Current Indications, Limitations and Future Developments

**DOI:** 10.3390/toxins4100913

**Published:** 2012-10-19

**Authors:** Sheng Chen

**Affiliations:** Department of Applied Biology and Chemical Technology, The Hong Kong Polytechnic University, Hung Hom, Kowloon, Hong Kong; Email: sheng.chen@polyu.edu.hk; Tel.: +852-3400-8795; Fax: +852-2364-9932

**Keywords:** Botulinum neurotoxin, clinical indications, future developments, novel applications

## Abstract

Botulinum neurotoxins (BoNTs) cause flaccid paralysis by interfering with vesicle fusion and neurotransmitter release in the neuronal cells. BoNTs are the most widely used therapeutic proteins. BoNT/A was approved by the U.S. FDA to treat strabismus, blepharospam, and hemificial spasm as early as 1989 and then for treatment of cervical dystonia, glabellar facial lines, axillary hyperhidrosis, chronic migraine and for cosmetic use. Due to its high efficacy, longevity of action and satisfactory safety profile, it has been used empirically in a variety of ophthalmological, gastrointestinal, urological, orthopedic, dermatological, secretory, and painful disorders. Currently available BoNT therapies are limited to neuronal indications with the requirement of periodic injections resulting in immune-resistance for some indications. Recent understanding of the structure-function relationship of BoNTs prompted the engineering of novel BoNTs to extend therapeutic interventions in non-neuronal systems and to overcome the immune-resistance issue. Much research still needs to be done to improve and extend the medical uses of BoNTs.

## 1. Introduction

Botulinum neurotoxins (BoNTs) cause flaccid paralysis by interfering with vesicle fusion and neurotransmitter release in neuronal cells [1,2,3]. BoNTs are 150 kDa di-chain proteins with typical A-B structure-function properties, where the B (binding) domain binds to surface components on the mammalian cell and translocates the A (active) domain to an intracellular location [4,5]. BoNTs are organized into three functional domains: an N-terminal catalytic domain (light chain, LC), an internal translocation domain (heavy chain, HC_T_), and a C-terminal receptor binding domain (heavy chain, HC_R_) [6]. There are seven serotypes of BoNTs termed A–G. Each serotype of BoNT cleaves one of the SNARE proteins, VAMP2, SNAP25 or syntaxin 1a [7]. These SNARE proteins mediate mammalian neuronal exocytosis, therefore BoNTs cause flaccid paralysis by inhibiting exocytosis and neurotransmitter release. Due to their high efficacy, tolerance, longevity and satisfactory safety profile, BoNTs are now the most widely used therapeutic proteins. This review describes the current clinical usage of BoNTs and the efforts to further extend and optimize their value in therapeutic interventions. 

## 2. Mechanisms of Action of BoNTs

**Figure 1:**
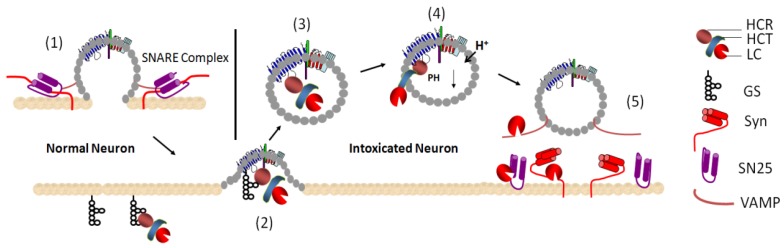
Multiple-step process of neuron intoxication by BoNTs. (**1**) In a normal neuron, neurotransmitter release is mediated by vesicle exocytosis facilitated by SNARE complex; (**2**) the HCR of BoNT binds gangliosides (GS) on the plasma membrane. Fusion of synaptic vesicles to the plasma membrane exposes loops of synaptic vesicles acting as BoNT protein receptor. The HCR binds to both GS and protein receptor simultaneously; (**3**) complexes of synaptic vesicle proteins are endocytosed to be recycled, which internalizes BoNT into neuronal cells; (**4**) the acidic environment triggers insertion of the HCT domain, which facilitates translocation of a partially unfolded LC (blue) through a channel made by the HCT (orange); (**5**) after translocation into the cytosal of a neuron, BoNT LC cleave SNARE proteins to inhibit exocytosis. Cleavage of VAMP2 and SNAP25 by LC/F and LC/A, respectively, is indicated. GS, gangliosides; Syn, syntaxin 1a; SN25, SNAP25; HC_T_, translocation domain (heavy chain); HC_R_, receptor binding domain (heavy chain); LC, light chain.

Whether absorbed directly from the GI track or injected into muscles, BoNTs are transported to neuromuscular junction (NMJ) areas. The initial step by which BoNTs interact with neuronal cells involves specific binding to the surface receptors through the HC_R_ domain of BoNTs. Complex gangliosides function as receptors for Clostridium Neurotoxins (CNTs) [8]. Different neurotoxins have been reported to interact with different types of gangliosides and GD1a and GT1b types for most of the BoNTs and Tetanus Neurotoxin (TeNT) [9,10,11,12,13,14,15]. Recent identification of synaptic vesicles as BoNT protein receptors advances the double-receptor theory of CNTs. SV2 was proved to be the receptor for BoNT/A, D, E and F; Synaptotagmin I (SytI) and/or Synaptotagmin II (SytII) were the receptors for BoNT/B, G and D-SA [16,17,18,19,20,21,22,23].This concept suggests that the HC_R_ domain of CNTs initially binds to abundant complex gangliosides to facilitate the anchoring of CNTs to the plasma membrane surface of neurons [24,25]. Simultaneous interactions with gangliosides and protein receptors may create a high affinity binding force, which may be critical for the subsequent endocytosis process of BoNTs (Figure 1) [8,24,25]. 

After specific receptor binding and internalization triggered by the vesicle recycling pathway, the CNTs are internalized into the vesicle of neuronal cells. Although the mechanism of toxin translocation is not fully understood, using single cell assay, a sequence of a progressive light chain BoNT/A (LC/A) translocation process is proposed. The charged surface of the translocation domain of BoNT/A inserts into the lipid bilayers and forms the channel; the belt region of the BoNT/A may act as a chaperon for LC unfolding and drags the unfolded LC through the HC channel. This is evidenced by the transient occluded conductance indicating the transit process, followed by the non-occluded transductance indicating the completion of the translocation. The dissociation of LC from HC then occurs by breaking the disulfide bond, which may be triggered by the neutral pH and the reducing environment at the cytosol, promoting the release of LC from the HC (Figure 1) [26]. A lot of questions remain, for example, the mechanistic details of the translocation process, such as the function of belt, how the unfolding and refolding process is regulated, and the fate of the HC channel. More work needs to be done to better understand the physiology of this most important process in various AB toxins [27,28].

**Figure 2:**
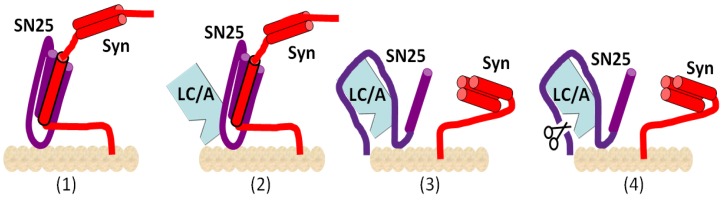
High affinity binding of LC/A to SNAP-25. Schematic of LC/A interactions with membrane-bound SNAP-25. (**1**) At the cell plasma membrane, syntaxin 1a (*Syn*) and SNAP-25 form a SNARE complex; (**2**) The N terminus of LC/A binds residues 80–110 of SNAP-25; (**3**) which facilitates substrate binding in competition with syntaxin 1a; (**4**) After SNAP-25 cleavage, LC/A has a high affinity for membrane-bound SNAP-25(1–197).

After being translocated into the cytosol of neuronal cells, the LCs of CNT in the cytosol cleave SNAP (Soluble NSF Attachment Protein) Receptor (SNARE) proteins, leading to inhibition of the exocytosis of neurotransmitter-carrying vesicles. Current studies suggest that translocated LC of BoNT may follow a certain trafficking pathway to reach its substrate. It was shown that LC/A displayed membrane localization in a neuronal cell, yet LC/E did not show plasma membrane association as strong as LC/A. Nevertheless, fractionation experiments with the transfected LC/E in neuronal cells indicated that LC/E was also membrane associated [29,30]. Further studies showed that LC/A membrane localization was due to the high affinity binding to its substrate SNAP25 that is membrane localized. The high affinity binding of LC/A to SNAP25 was through two discontinuous interaction sites: one through the interaction of the N-terminus of LC/A to the (80~110) region of SNAP25 and the other through the binding of SNAP25(141–206) to the substrate binding cleft of LC/A (Figure 1) [31]. Since biologically functional SNAP25 is always associated with snytaxin or formation of SNARE complex (SNAP25, Syntaxin and VAMP) on the plasma membrane, the binding of the N-terminus of LC/A to the available region of SNAP25 (80–110) in the SNARE complex helps the LC/A to compete for the subsequent substrate binding and cleavage (Figure 2). 

There are seven serotypes of BoNTs (termed A–G) that cleave specific residues on one of the three SNARE proteins: serotypes B, D, F, and G cleave VAMP-2, serotypes A and E cleave SNAP25, and serotype C cleaves SNAP25 and syntaxin 1a [4]. Each LC cleaves one of the SNARE proteins except for LC/C, which cleaves both SNAP25 and syntaxin 1a. The crystal structures of all LCs of BoNT have been resolved and have been shown to have a very similar structural confirmation with a Zn ion coordinated to the active sites of the LCs [32,33,34,35,36,37]. In contrast to other metalloproteases such as thermolysin, biochemical characterization of LC substrate indicated that BoNT LCs require a long substrate for efficient cleavage [38,39,40]. 

**Figure 3:**
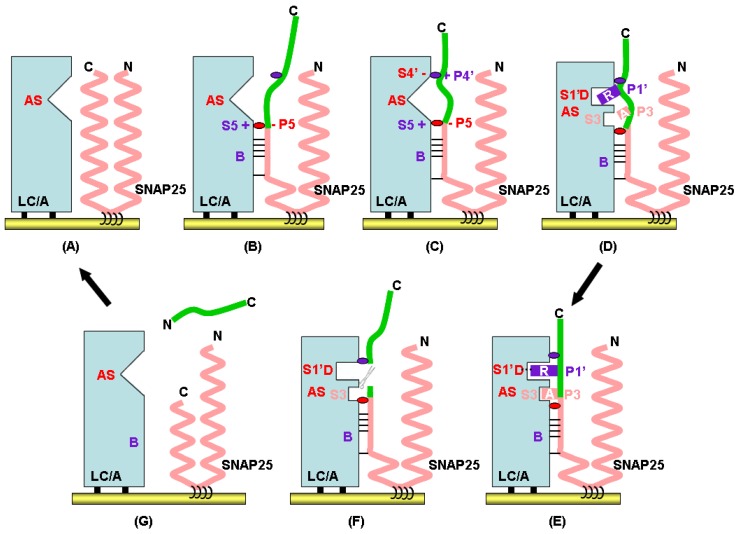
Multistep recognition and cleavage of SNAP25 by LC/A. (**A**) and (**B**), at the plasma membrane LC/A initially binds to SNAP25 through discontiguous surface interactions between residues within the belt region of LC/A and the B region residues of SNAP25. The Velcro-like binding of SNAP25 to LC/A aligns the P5 residue Asp^193^ to form a salt bridge with Arg^177^, an S5 pocket residue, at the periphery of one side of the active site; (**C**), this orientates SNAP25 for the formation of a salt bridge between the P4'-residue Lys^201^ and the S4'-residue LC/A(Asp^257^); (**D**), these interactions broaden the LC/A active site cavity and dock Arg^198^, the P1'-residue, via electrostatic interactions with Asp^370^ within the S1'-pocket. The fine-tuning of the alignment of Arg^198^ into the S1'-pocket is facilitated by the binding of SNAP25-Ala^195^ to P3 residues in the hydrophobic S3 pocket of LC/A. The proper alignment of the P1'-P3 sites into the Zn^2+^ active motif (**E**) facilitates the substrate cleavage (**F**). After cleavage, the C-terminal product dissociates from LC/A, which returns the AS to the original conformation (**G**).

Decoding the complex structures of LC/A-SNAP25 and LC/F-VAMP2 depicted the mode of LC recognition of their substrates, SNAP25 or VAMP2 [41,42]. The recognition of SNAP25 by LC/A is mediated by exosites: the α-exocite binding is initiated by substrate binding distal to the AS and recognition of the SNAP25 scissile bond by the AS of LC/A. Further biochemical studies resolved the step-by-step binding and recognition of SNAP25 by LC/A [43]. The mechanism of LC/A recognition and cleavage of SNAP25 involves sequential steps representing SNAP25 recognition and active site organization. Initial interactions involve discontinuous surfaces between residues within the belt region of LC/A and the substrate binding (B) region residues of SNAP25. The Velcro-like binding of SNAP25 to LC/A aligns the P5 residue Asp^193^ to form a salt bridge with Arg^177^, an S5 pocket residue at the periphery of one side of the active site. Although the exact order of each step of recognition of SNAP25 by BoNT/A at the active site is not clear, the initial binding could subsequently orientate SNAP25 for the formation of a salt bridge between the P4'-residue SNAP25(Lys^201^) and the S4'-residue LC/A(Asp^257^). These interactions broaden the LC/A active site cavity and dock Arg^198^, the P1'-residue, via electrostatic and hydrophobic interactions within the S1'-pocket. The fine tuning of the alignment of Arg^198^ into the S1'-pocket resulting in the precise alignment of the scissile bond is facilitated by the binding of the P3 residue, SNAP25-Ala^195^, into the hydrophobic S3 pocket of LC/A. The proper docking of the P1'-P1 sites into the AS site initiates substrate cleavage. After cleavage, the P4'-residue dissociates from the S4'-residue of LC/A, which converts the AS into a smaller conformation, facilitating dissociation of the P1'-residue from the AS (Figure 3) [43].

**Table 1:** Current indications for BoNT based therapies.

Status	Indications	BoNT product (Year of approval)	Remarks
**FDA approved indications**	Strabismus	Oculinum/BOTOX (1989)	Very effective but repetitive injections are required, therefore more suitable for temporary uses
	Blepharospasm	Oculinum/BOTOX (1989)	Very effective and no more trials are required
	Hemifacial spasm	Oculinum/BOTOX (1989)	Very effective and no more trials are required
	Cervical dystonia	BOTOX (2001), Dysport (2009), Xeomin (2010), NeuroBloc (2000)	Very effective but larger doses may be needed, therefore immune-resistance might sometimes develop in some patients
	Cosmetic use	BOTOX (2000, Canada)BOTOX (2012, US)	Very effective and safe for long-term use
	Axillary hyperhidrosis	BOTOX (2001, UK and Canada), BOTOX(2004, US)	Effective and safe, but painful at the injection sites
	Chronic migraine	BOTOX (2010)	Safe and effective for randomized studies but not placebo controlled trials
	Neurogenic detrusor overactivity	BOTOX (2012)	Remarkable efficacy and minimal side effects
**Off-labeled use indications**	Lower urinary tract disorders	BOTOX	Remarkable efficacy and minimal side effects
	Gastrointestinal tract disorders	BOTOX	Commonly used for some indications, but effects are relatively short-lived
	Spasticity	BOTOX	Can be considered as a first-line treatment, but should be used at the early stage
	Spasmodic dysphonia	BOTOX	Effective but more controlled studies are needed
	Sialorrhea	BOTOX	Effective on reducing excessive salivation but effective therapeutic dosages and the ideal form of application remain to be established
	Temporomandibular disorder	BOTOX	Correct injection technique and appropriate dosing guidelines are very important for successful results
	Chronic musculoskeletal pain	BOTOX	Effective for some patients who have not responded favorably to first-line treatments
	Vaginism	BOTOX	Effective but reports are limited
	Wound healing	BOTOX	Improvement of wound healing
	Diabetic neuropathy	BOTOX	Effective and safe treatment but reports are limited

## 3. Clinical Applications of Botulinum Neurotoxins

BoNT intoxication is reversible and muscles will function again upon clearance of BoNT from the affected neuronal cells. In addition, local application of BoNT largely limits BoNT toxicity to the applied area and does not spread to the central neuron or if it does then only really slowly; the action of BoNT can last for up to six months, thus frequent applications are not needed. These features of BoNT have turned it from a deadly agent into use for novel therapies for a range of neuromuscular conditions [44,45,46,47,48,49,50,51,52,53]. BoNT/A was approved by the U.S. FDA to treat strabismus, blepharospam, and hemifacial spasm as early as 1989 and then for treatment of cervical dystonia, glabellar facial lines, axillary hyperhidrosis, chronic migraine and for cosmetic use (Table 1). The efficacy of BoNT/A in treating dystonia and other disorders related to involuntary skeletal muscle activity, coupled with the satisfactory safety profile, has prompted its empirical/off-label use in a variety of ophthalmological, gastrointestinal, urological, orthopedic, dermatological, secretory, and painful disorders (Table 1). There are three preparations of BoNT/A, namely BOTOX (onabotulinumtoxinA, Allergan, approved by FDA in 1989), Dysport (abobotulinumtoxinA, Medicis, approved by FDA in 2009) and Xeomin (incobotulinumtoxinA, Merz, approved by FDA in 2010). On December 11, 2000, a Botulinum Neurotoxin serotype B product (MYOBLOC^™^) was approved by the FDA in the United States as a treatment for patients with cervical dystonia to reduce the severity of abnormal head position and neck pain associated with cervical dystonia. MYOBLOC is the U.S. trade name for Solstice Neurosciences' Botulinum Neurotoxin serotype B product. This product also received marketing authorization from the Committee for Proprietary Medicinal Products of the European Union and is available there under the name Neurobloc. The duration of effect is approximately 12 to 16 weeks, a little shorter than that of BoNT/A [54,55,56]. 

### 3.1. FDA Approved Clinical Use of BoNTs

Alan Scott and Edward Schantz were the first scientists to apply BoNT for therapeutic use. By 1973, Scott used BoNT/A in a monkey experiment and in 1980 he used the toxin to treat strabismus in humans for the first time. Since then the application of BoNT forn different kinds of neuronal disorders has been widely explored. The U.S. FDA has approved the use of BoNTs for several indications that were proven to be effective and safe [57,58,59]. 

#### 3.1.1. Strabismus

In 1989, the U.S. Food and Drug Administration (FDA) approved the use of BoNT/A (oculinum) to treat strabismus. The product’s name was later changed to BOTOX. Further research also indicated its effectiveness in most strabismus patients. However, due to the requirement of repeated injections, BoNT/A may not be considered as an alternative to surgery [60,61,62,63]. It is therefore more suitable for temporary use or in cases where surgical procedure is undesirable. Long-term treatment with BoNT/A has also proven to be safe and efficient [62,64]. 

#### 3.1.2. Blepharospasm

In 1989, the U.S. FDA approved the use of BoNT/A for blepharospasm treatment. Further studies have suggested that over 90% of patients who received BoNT/A treatment alone showed sustained improvement in their disease onset and the safety profile of BoNT/A treatment on blepharospasm patients is excellent [65,66]. It was also suggested that there is no need to conduct placebo-controlled trials even though so far information on the efficiency of randomized and controlled studies is lacking. 

#### 3.1.3. Hemifacial Spasm

In 1989, the U.S. FDA approved the use of BoNT/A to treat hemifacial spasm. A large open, case-controlled study showed that over 76% of the patients benefited from BoNT/A treatment [67,68,69]. A mega analysis that reviewed most of the reported studies suggested that no new large placebo-controlled trials were needed, even though only one placebo-controlled trial had been reported to date [69]. 

#### 3.1.4. Cervical Dystonia

The first placebo controlled trial of using BoNT/A in CD treatment was conducted in 1987 and showed promising results regarding the improvement of disease onset [70]. Subsequent case studies and placebo-controlled studies indicated that over 70% of the patients who received BoNT/A treatment showed significant improvement in their symptoms and pain relief and the beneficial effect normally lasts around 12 weeks [71,72,73,74,75]. On December 21, 2000, BoNT/A received FDA approval for treatment of cervical dystonia. In addition to BOTOX, there are two other forms of BoNT/A products, Dysport and Xeomin, and BoNT/B (MYOBLOC^™^ or NeuroBloc) that are also approved by FDA to treat cervical dystonia. There are no significant differences in terms of their efficacy in cervical dystonia treatment, although BoNT/B shows generally higher immunogenicity in patients. The greater benefit of BoNT/B treatment is for BoNT/A-resistant patients [76,77,78,79,80]. 

#### 3.1.5. Cosmetic Use

In 2000, Canada approved the use of BOTOX for focal muscle spasticity and cosmetic treatment of wrinkles at the brow line. On April 15, 2002, the U.S. FDA announced the approval of the use of BOTOX for cosmetic uses. Recent BoNT/A indications for cosmetics applications include glabellar frown lines, horizontal forehead lines, crow’s feet, bunny lines, perioral lines, mental crease and dimpled chin, mouth frown, platysmal bands and horizontal neck lines. 

#### 3.1.6. Axillary Hyperhidrosis

In 2001, the United Kingdom and Canada approved the use of BOTOX for axillary hyperhidrosis (excessive sweating). In July 2004, the FDA approved the use of BOTOX to treat severe axillary hyperhidrosis that cannot be managed by topical agents, such as prescription of antiperspirants. In addition to axillary hyperhidrosis, BoNT/A is also clinically used to treat palmar hyperhidrosis. One of the most troublesome disadvantages associated with this therapy is pain at the injection sites. It has been suggested that the reconstitution of botulinum toxin A in a solution of lidocaine could be an easy alternative procedure to reduce the discomfort associated with these injections [81,82].

#### 3.1.7. Chronic Migraine

Randomized, double-blinded, placebo-controlled trials suggested that BoNT/A was effective in the treatment of chronic migraine prophylaxis, although the gain over placebo was modest [83,84,85]. Still, the excellent tolerability of BoNT/A makes it an extremely attractive alternative for patients who fail to tolerate, and therefore discontinue traditional oral prophylactics. Although the mechanisms through which BoNT/A may exert its benefit remain unknown, BoNT/A is a welcome addition to the available treatment options for chronic migraine, which is often a disabling and difficult-to-manage condition. In 2010, the U.S. FDA approved the use of BoNT/A to treat chronic migraine. 

#### 3.1.8. Detrusor Overactivity

One of the most widespread urological applications of BoNT/A is the treatment of detrusor overactivity (DO), which is characterized as a major pathology underlying urge urinary incontinence and urgency-frequency syndromes. Several multicenter randomized controlled trials involving more than 600 patients showed notable improvement of incontinence in most of the neurogenic detrusor overactivity (NDO) patients [86,87,88,89,90]. Another major benefit of BoNT/A treatment is the reduction in urinary infections such as pyelonephritis, orchitis, and prostatitis commonly observed in NDO patients [88,91]. The average duration of effect is about a year and adverse side effects are rare. Allergan’s Phase III clinical trial on detrusor overactivity associated with neurologic conditions such as multiple sclerosis (MS) or spinal cord injury (SCI) also showed significant reductions in the frequency of urinary incontinence episodes in patients treated with BOTOX. In 2012, the US FDA approved BOTOX^® ^(onabotulinumtoxinA) for injection for the treatment of urinary incontinence due to detrusor overactivity associated with neurologic conditions e.g. SCI and MS in adults who have an inadequate response to or are intolerant of an anticholinergic medication.

### 3.2. Off-Labeled Use of BoNTs

In addition to FDA approved indications, BoNT/A has been empirically used in a variety of urological, ophthalmological, gastrointestinal, orthopedic, dermatological, secretory, and pain disorders. Indications are grouped into different categories and discussed as follows.

#### 3.2.1. Lower Urinary Tract Disorders

In addition to FDA approved urinary tract disorder, detrusor overactivity, other lower urinary tract disorder indications for BoNT/A include detrusor sphincter dyssynergia, painful bladder syndrome and prostatic obstruction. BoNT/A treatment of lower urinary tract disorders has remarkable efficacy and minimal side effects and thus will be a key future treatment option; meanwhile, more controlled trials are needed to test the feasibility of various treatment options [92,93,94,95,96]. 

#### 3.2.2. Gastrointestinal Tract Disorders

The most common GI tract indications with the best available data are achalasia, anal fissures and gastroparesis. In other GI tract disorders, such as esophageal spasm, sphincter of Oddi dysfunction and anismus as well as obesity, only preliminary data are available. In conclusion, for GI tract disorders, the concern remains that the effects of BoNT/A are relatively short-lived and definitive treatment is delayed [97,98,99,100,101,102,103]. Additional trials are necessary to assess its efficacy duration of action, and for comparison with other therapeutic agents. 

#### 3.2.3. Spasticity

Spasticity is characterized by upper motor neuron dysfunction and if severe, can lead to considerable motion restriction and eventually serious disability. Spasticity may result from various etiologies including stroke, spinal cord injury, multiple sclerosis, traumatic brain injury, cerebral palsy, and neurodegenerative diseases such as Parkinson’s disease. Available therapeutic interventions for spasticity are often of limited benefit. BoNT/A can be considered a first-line treatment for focal or multifocal spasticity. It should be used at an early stage to prevent soft tissue shortening from occurring as a result of the combined effect of spasticity and limb immobility [104,105,106,107,108,109]. BoNT/A shows a good safety profile and the side effects are minimal and rare in spasticity patients [110].

#### 3.2.4. Spasmodic Dysphonia

Spasmodic dysphonia (SD) is a focal laryngeal dystonia characterized by involuntary action-induced spasm of the muscles that control vocal fold motion and adductor spasmodic dysphonia or (ADSD) is the most common presentation. Currently available pharmaceutical agents have the shortcomings of partial efficacy, unwanted adverse effects and drug interactions. BoNT/A has been established as an effective treatment for ADSD based on one convincing randomized double-blind cross-over, placebo-controlled study and three double blind, placebo-controlled/randomized group cohort studies [111,112]. In conclusion, BoNT/A treatment of ADSD is safe and highly tolerated. Side effects are minimal and rare. 

#### 3.2.5. Sialorrhea

Sialorrhea or excessive salivation and drooling are common disabling manifestations found in different neurological disorders. Sialorrhea is most commonly associated with infant cerebral palsy, Parkinson’s disease and amyotrophic lateral sclerosis [113,114]. BoNT/A can reduce excessive or uncontrolled salivation via autonomic denervation rather than muscle denervation. The effective therapeutic dosages and ideal form of application remain to be established, and require further controlled clinical trials involving larger sample sizes. More research effort is needed to determine the ideal dosages and injection location, as well as to improve the technique of BoNT/A injections. BoNT/B (Neurobloc) has also been used to treat sialorrhea related to different kinds of diseases and showed very high efficacy, tolerability and safety profile. When compared to BoNT/A, BoNT/B treatment shows a slightly shorter duration of action and a higher dose requirement, while it is more efficient and cost effective [115,116,117,118].

#### 3.2.6. Temporomandibular Disorder

Temporomandibular disorders (TMD) are a set of musculoskeletal dysfunctions within the masticatory system with multiple etiologies. TMD may be associated with headache, periauricular pain, neck pain, decreased jaw excursion, locking episodes, and noisy joint movement. BoNT/A has been used to treat TMD since 1999 [119]. BoNT/A injections produced a statistically significant improvement in pain, function, mouth opening, and tenderness. Due to the complex nature of TMDs and the proximity of affected muscles to facial nerves, correct injection technique and appropriate dosing guidelines are very important for successful results. Further studies are needed to optimize the current procedure of BoNT/A treatment. 

#### 3.2.7. Chronic Musculoskeletal Pain

Chronic musculoskeletal pain is due to musculoskeletal disorders in various locations in the human body. BoNT/A has been successfully used in the treatment of spasmodic torticollis, limb dystonia, and spasticity. Investigators have thus become interested in its potential use in treating many chronic pain conditions. There is strong evidence supporting the use of BoNT/A as a treatment for pelvic pain, plantar fasciitis, temporomandibular joint dysfunction associated facial pain, chronic lower back pain (LBP), carpal tunnel syndrome, joint pain, in complex regional pain syndrome and in selected neuropathic pain syndromes [120,121]. It does appear that BoNT/A is useful to certain patients, especially those patients who have not responded favorably to first-line treatments, thus providing a window of therapy, while its duration of action may exceed that of conventional treatments. This seems a promising treatment that must be further evaluated.

#### 3.2.8. Other Indications

BoNT/A was also tried in several other less common conditions such as vaginism, wound healing, and diabetic neuropathy. To date, only a few reports regarding the use of BoNT/A in the treatment of patients with vaginismus have been published. Almost all patients showed positive response to BoNT/A treatment [122,123]. Wounds of the face, especially those lying perpendicular to the lines of Langer, are known to heal poorly with conspicuous scarring. A double-blind, randomized study showed the improvement of wound healing after hemorrhoidectomy [124]. BoNT/A was also effective for forehead wound healing and ugly scars of the face [125,126]. BoNT/A was thought to decrease the expression of SP, CGRP, TGF-beta1, and alpha-SMA in wound healing in a dose-dependent manner with no effect on the healing time [127]. Some researchers are testing the effect of BoNT/A on urethral wound healing and collagen deposition in hypertrophic scars using animal models [128,129]. BoNT/A treatment for different types of diabetic neuropathy has been reported [130,131,132,133,134,135]. Although the data are still very limited, all case studies supported BoNT/A as an effective and safe treatment. Further randomized and case studies will be needed to gain more insight into the application of BoNT/A to treat diabetic neuropathy. 

## 4. Limitations of Currently Available BoNT Therapies and Novel Product Development

The unique characteristics and pharmacological properties of BoNT have made it a versatile treatment option for a growing number of indications. Currently available BoNT therapies have certain limitations. These include but are not limited to the optimization of current therapies including choices of dosage, injection sites, injection techniques, injection interval, development of different types of BoNT/A products with different pharmacological properties, exploration of new indications for BoNT, future development for treatment of neuronal disorders such as chronic pain, novel BoNT to treat central nervous system disorders, standardization of off-labeled uses of BoNT and designation of more randomized, placebo controlled trials, investigation of mechanisms of anti-nociceptive activity of BoNT and its application to analgesia uses, development of novel BoNT-based products to control non-neuronal hypersecretion diseases, development of BoNT products with lower immunogenicity, and exploration of other BoNT serotypes as therapeutic agents. 

This review focuses on the knowledge on the development of novel BoNT to extend its therapeutic applications and the immune-resistance issue of current therapies. Clinical use of BoNTs is limited to conditions that affect neuromuscular activity [46,52] due to the neuronal tropism of BoNT: the recognition of a neuronal specific cell surface receptor and neuronal SNARE protein as substrates. Non-neuronal SNARE isoforms are involved in divergent cellular processes that include fusion reactions in cell growth, membrane repair, cytokines and synaptic transmission [136]. For example, SNAP23 complexes with non-neuronal VAMP and syntaxin isoforms mediate non-neuronal vesicle exocytic processes, including the secretion of airway mucus, antibodies, insulin, gastric acids, and ions [137,138,139,140,141,142,143,144,145,146,147,148]. Targeting SNAP23 with a modified BoNT may reduce the secretion processes of hypersecretion syndromes. 

The therapeutic benefits of BoNT for treatment of conditions associated with involuntary muscle spasm and contractions, for cosmetic use, and other applications are transient and repeated injections are necessary. In some patients, BoNT could elicit neutralizing antibodies against the corresponding toxin, thus reducing the beneficial effects or rendering the patient completely unresponsive to further treatment [44,49,149,150,151,152,153,154,155]. The exact percentage of patients who may develop immuno-resistance to BoNT treatment is unknown, but it is commonly believed that there are fewer patients who develop blocking antibodies when treated with BoNT/A than with BoNT/B [49,155]. This is probably due to the use of lower doses of BoNT/A complex than BoNT/B complex [156,157]. The development of blocking antibodies is also more common in patients who receive treatment for cervical dystonia or spasticity, which requires larger doses and periodic administration of toxin, while it is less common in patients who are treated for laryngeal dystonia, blepharospasm or cosmetic use, all of which require smaller doses for treatments [49,158,159]. Thus reducing the treatment dose may help to reduce the development of immuno-resistance.

### 4.1. Engineering Novel BoNT to Target non-Neuronal Substrate

Attempts have been made to engineer novel BoNT derivatives, and one of them is the development of therapeutic proteins that possess the endopeptidase activity of BoNT, with different cellular specificity but without the inherent toxicity of neurotoxins. By replacing the HCR of BoNT with a new peptide or protein-targeting domain, the resulting chimeric protein can be retargeted to a new cell type defined by the new binding domain. This includes the NGF-LHCT/A and wheat germ agglutinin-LHCT/A for both neuronal and non-neuronal cells. A therapeutically relevant application is to target ECL (Erythrinacristagalli lectin)-LHCT/A conjugate to nociceptive afferents and airway epithelium cells [146,160,161,162,163,164]. Retargeting BoNT to non-neuronal system such as airway epithelium cells has been limited due to selective cleavage of neuronal-specific SNARE proteins, SNAP25, VAMP-2 and syntaxin 1a by the catalytic domain of BoNT. Therefore, the development of non-neuronal SNARE protein specific LC is a prerequisite to develop novel BoNT for non-neuronal indications. 

**Figure 4:**
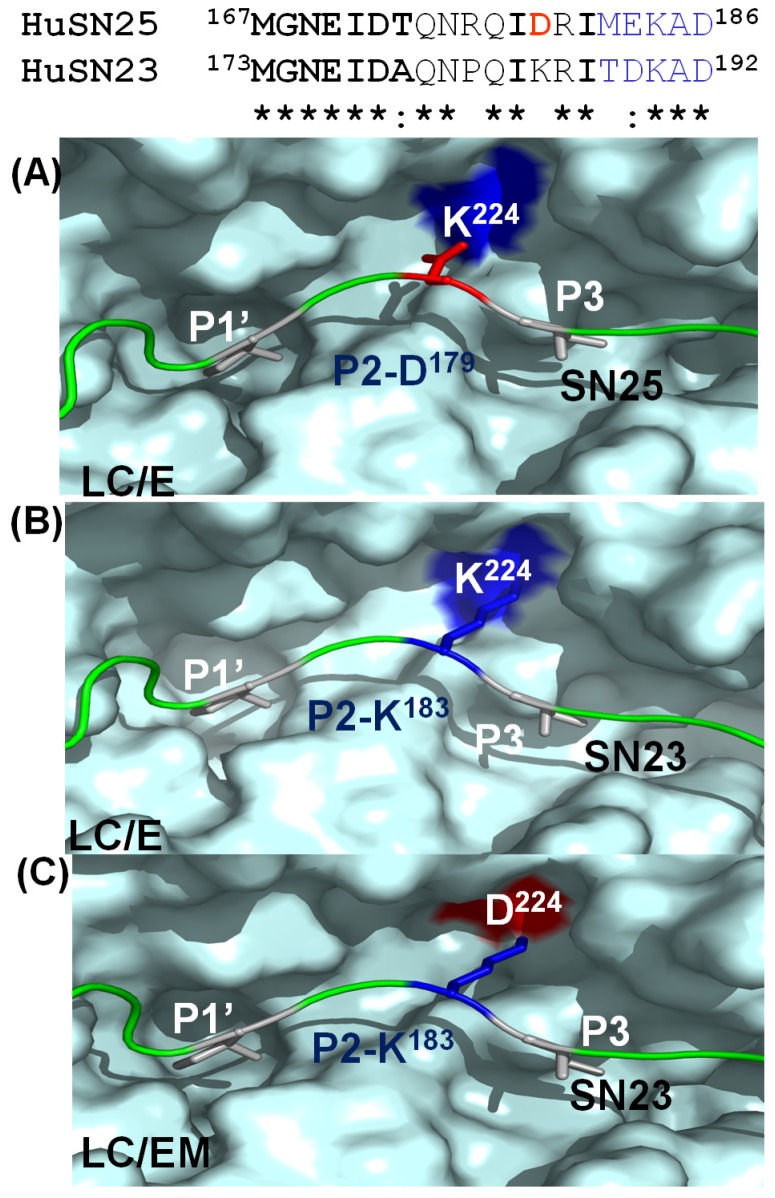
K^185^ of human SNAP23 contributes to substrate recognition by BoNT/E. (**A**) Recognition of D179 of SNAP25 by S2 pocket, K224 of LC/E; (**B**) inability to cleave SNAP23 by LC/E was due to the impairment of P2-S2 recognition; (**C**) Cleavage of SNAP23 by LC/E (K^224^D) by fine tuning the P2-S2 interaction.

The retargeting of the catalytic activity of BoNTs to non-neuronal SNARE isoforms has been investigated [165]. SNAP23 is a non-neuronal isoform of SNAP25 and it mediates the process of different secretion events in non-neuronal systems such as airway mucus, gastric acid and antibody secretion. Excessive secretion of these substances is associated with different diseases such as asthma, chronic obstructive pulmonary disease, gastric efflux, diabetes and inflammatory and immune disorders. Targeting SNAP23 with a novel BoNT derivative may reduce the secretion processes of hypersecretion syndromes. A mutated BoNT/E light chain, LC/E(K^224^D), was engineered and showed extended substrate specificity to cleave SNAP23 and the natural substrate, SNAP25, but not SNAP29 or SNAP47. Upon direct protein delivery into cultured human epithelial cells, LC/E(K^224^D) cleaved endogenous SNAP23 so as to inhibit the secretion of mucin and IL-8 [165] (Figure 4). These studies show the feasibility of genetically modified LCs to target a non-neuronal SNARE protein, which will extend the therapeutic potential of BoNT for treatment of human hypersecretion diseases.

### 4.2. Combating BoNT Immunoresistance Issues

One way to overcome the immuno-resistance problem in BoNTs is to engineer more active BoNTs, which will reduce the amounts of protein required for therapy and may decrease the development of immune response to the therapy. Rumme, *et al.* modified a ganglioside binding motif of the HC domain of BoNT/A that enhances the binding and toxicity up to three-fold relative to the wild type toxin [166,167]. However, the engineering of BoNT through modification(s) of its receptor binding sites may affect the selectivity of the binding event and protection by current vaccine derived from the HCs of BoNTs. In addition, the modification of binding site(s) may not successfully increase the potency enough to prevent the development of immuno-resistance. Modification of LC to alter its activity may well be a better way to achieve this goal. 

**Figure 5:**
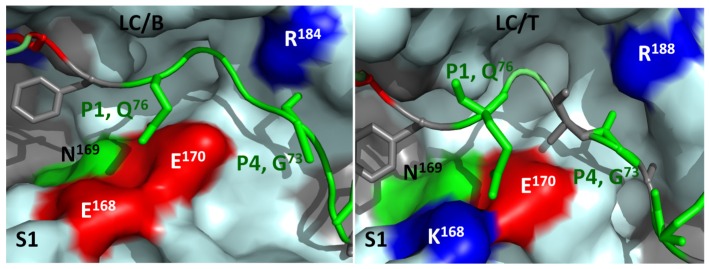
Optimization of LC/T substrate recognition pockets increases its catalytic activities. The S1 pocket of LC/B and LC/T formed by residues E^168^N^169^E^170^ and K^168^N^169^E^170^, respectively, recognizes P1, Q^76^ of VAMP2. Mutation LC/T K^168^E increases *k_cat_* by ~8 fold. The S4 pocket of LC/B and LC/T involves the residues R^184^ and R^188^. The mutation LC/T R^188^M increases *k_cat_* by ~5 fold.

A comparative study was conducted for LC/B and LC/T to provide proof of principle to engineer LC of CNTs with elevated activities. The comparative study of LC/B and LC/T substrate recognition enabled us to find that the S1 pocket mutation LC/T(K168E) increased the rate of native VAMP2 cleavage approaching the rate of LC/B, which explains the molecular basis for the lower kcat that LC/T possesses for VAMP2 cleavage relative to LC/B (Figure 5). In addition, R188M, a S4 pocket mutation, increased LC/T substrate hydrolysis by ~5 fold [168]. In LC/A, mutation of an active site of LC/A, K165L, resulted in a 4-fold increase in substrate hydrolysis [169]. These results suggest the possibility to achieve BoNT with higher activity through LC engineering. This analysis explains the molecular basis underlying the VAMP2 recognition and cleavage by LC/B and LC/T and provides insight into the possibility of extending the pharmacologic utility of these neurological reagents. Our further characterization of both LC/B and LC/T enabled us to engineer a LC/B derivative with 10~15-fold increase of catalytic activity. This novel LC/B has the potential for future therapy development [170].

The other way to counter the immuno-resistance issue of BoNT therapy is to block the epitopes on the BoNTs that are involved in neutralizing antibody production. By reacting the neutralizing antibodies from resistant patients to different domains of BoNT/A and B, a series of regions that may be involved in neutralizing antibody production have been identified [171,172,173]. It was reported that autoantibody responses could be suppressed against a selected epitope on a self-antigen by treatment with a monomethoxypolyethylene glycol (mPEG) of the epitope [174]. Therefore using a synthetic mPEG-peptide conjugate for a predetermined epitope could lower the titer of antibody response. Regions on the HC/B that showed strong immuno-response were conjugated to mPEG and pre-immunized mice before the administration of BoNT/A. It was shown that some of the mPEG conjugated peptides could actually reduce the neutralizing antibody production [175]. This result suggests that the tolerization procedure might be potentially useful for clinical applications for immuno-resistant patients.

## 5. Persistence of BoNT

One of the unique features of BoNTs is their longevity of action in neuronal cells. The longevity of action varies within different serotypes of BoNTs. The action of BoNT/A can last more than six months, while that of BoNT/E remains only up to four weeks. The mechanism of the longevity of action of BoNTs is not conclusive, although a few elegant reports have advanced our understanding in this field. It was hypothesized that membrane localization of LCs may contribute to their longevity, since LC/A with the longest activity in cells shows plasma membrane localization, while LC/E with the shortest activity shows less plasma membrane localization [176]. This hypothesis could not be proven by convincing research data. However, another study showed that LC membrane localization does not contribute to the longevity of LCs and that instead LC longevity in cells is due to their different protein degradation pathways [30]. LC/E has been shown to be associated with TRAF2, a RING finger protein implicated in ubiquitylation that promotes fast degradation of recombinant LC/E in neuronal cells [30]. In addition, the targeting of LC/A to similar ubiquitylation through a chimeric substrate, TRAF2- non-cleavable SNAP25, dramatically reduces its duration in a cellular model for toxin persistence. Differential susceptibility of the catalytic LCs to ubiquitin-dependent proteolysis therefore might explain the differential persistence of BoNT serotypes [30]. Another interesting research in Dolly’s group showed that two leucine residues near the C terminus of the protease light chain of BoNT/A (LC/A) contributed significantly to the persistence of action of BoNT/A while hese two leucine residues were mutated, its inhibition of exocytosis become shortened. The dileucine at the C-terminus of BoNT/A may contribute to its stability in the cells. In addition, genetically fusing LC(E) to a BoNT(A) enzymically inactive mutant (BoTIM(A)) yielded a novel LC(E)-BoTIM(A) protein that targets neurons, giving a potent and persistent cleavage of SNAP-25 with associated neuromuscular paralysis. Mutation of these two leucine residues in LC/A in LC(E)-BoTIM(A) gave transient neuromuscular paralysis similar to BoNT(E), reaffirming that these residues are critical for the persistent action of LC(E)-BoTIM(A) as well as BoNT(A) [177]. These two studies addressing the potential mechanisms of BoNT persistence are not conflicting, although it will be interesting to see whether the two dilucines contribute to the dissociation of BoNT/A to TRAF2. Further studies are needed to depict the molecular mechanism of persistence of BoNT.

## 6. Conclusion

BoNTs, in particular BoNT/A and B, have been successfully used to treat a large number of neuronal disorders through exploitation of their potential in interfering with a wide spectrum of physiological functions ranging from reduction of muscular contraction to pain alleviation. Their unique characteristics and pharmacological properties have made BoNTs a versatile treatment option for a growing number of indications. The future of BoNTs in medical applications is bright, yet more research is needed to improve their medical uses. One of the most important areas is to test the use of the recombinant BoNT as a therapeutic agent since the future engineering of novel BoNTs couldl be built upon a recombinant BoNT. 
